# Enhancing General Surgery Clerkships: The Application and Value of Standardized Patient-Based Situational Simulation Teaching

**DOI:** 10.7759/cureus.60845

**Published:** 2024-05-22

**Authors:** Qin Sun, Yueshan Pang, Xu Liu, Ming He, Jing Dong, Jiebin Xie

**Affiliations:** 1 Discipline Inspection Commission Office, North Sichuan Medical College, Nanchong, CHN; 2 Department of Geriatrics, Central Hospital of Nanchong, The Second Clinical School of North Sichuan Medical College, Nanchong, CHN; 3 Department of Gastrointestinal Surgery, Affiliated Hospital of North Sichuan Medical College, Nanchong, CHN

**Keywords:** clinical internship, clinical competency, teaching effectiveness, standardized patient, scenario teaching

## Abstract

Objective: We explored the value of a standardized patient-based situational simulation teaching method in general surgery internships.

Methods: A prospective, single-blind, randomized controlled trial was conducted with clinical medicine undergraduates from the 2020 cohort of our university as subjects. These students were randomly divided into a traditional teaching (TT) group and a combined teaching (CT) group based on their internship schedules. The TT group followed the conventional teaching model, while the CT group engaged in the standardized patient-based situational simulation teaching method. The study compared differences in pre-internship theoretical scores, post-internship theoretical scores, medical record writing quality, and student satisfaction between the two groups.

Results: The CT group (n=108) significantly outperformed the TT group (n=104) in post-internship theoretical scores and medical record writing quality (all *P*<0.05) and showed marked improvement in stimulating students’ interest in learning (*P*=0.015), enhancing clinical diagnostic and treatment abilities (*P*<0.001), improving doctor‒patient communication skills (*P*<0.001), strengthening medical mission sense (*P*<0.001), reinforcing physicians’ sense of responsibility (*P*<0.001), and facilitating the application of learned knowledge (*P*<0.001). These differences were statistically significant.

Conclusion: The standardized patient-based situational simulation teaching method (CT) in general surgery internships has been highly recognized by students and can enhance their clinical competency, offering considerable value for broader.

## Introduction

Clinical internship is a crucial phase for medical students to transform their basic theoretical knowledge into clinical skills [1,2]. Traditional internship models are teacher-centered, where students passively receive information, with fewer opportunities for full-process participation in clinical practice, resulting in low learning interest [3]. In recent years, student-centered teaching models, such as problem-based learning (PBL), case-based learning (CBL), and team-based learning (TBL), along with the mixed use of standardized patients (SPs), have been advocated by students and teachers alike, shifting students’ learning approach from passive reception to active exploration [4]. However, these methods may not fully meet the needs of medical students in terms of transferring knowledge, doctor‒patient communication, and comprehensive judgment, with their limitations possibly stemming from the insufficient cultivation of role consciousness and responsibility [5].

In contrast, the situational teaching model breaks traditional constraints by engaging students in on-site scenario simulations, effectively improving their abilities in autonomous learning, problem-solving, and in-depth investigation of complex issues [6]. This method has proven to be highly effective in fields such as nursing. Additionally, the use of SPs effectively addresses the lack of diversity in case types and low patient cooperation in medical internships and has been widely applied in clinical teaching. Preliminary studies have shown that interaction with SPs allows students to gain a deeper understanding of disease characteristics and their influencing factors, significantly improving their communication skills and clinical thinking ability [7,8]. However, there is relatively little literature on whether integrating SPs with situational teaching can further enhance students’ clinical capabilities. Therefore, our study introduced a new SP-based scenario teaching model and explored its effects on clinical internships.

## Materials and methods

Study subjects and methods

Study Subjects

This research was designed as a prospective, single-blind randomized controlled trial involving clinical medicine undergraduates from the 2020 cohort at North Sichuan Medical College who were undergoing internships in the Gastrointestinal Surgery Department of the Affiliated Hospital from November to December 2023. The research methodology was approved by the Human Research Ethics Committee of the Affiliated Hospital of North Sichuan Medical College (Ethics Approval Number: 2023ER256-l). Using a random number table method, students were allocated to the combined teaching (CT) group, or the traditional teaching (TT) group based on different internship timings. The students in each group were divided into two teams (each with 5-6 students) with each assigned an instructor. Additionally, an SP was allocated to each team in the CT group.

Training of SPs and Selection

Volunteers who were third-year postgraduate students in surgery at our hospital were recruited as SPs, and our research group’s professor team was responsible for their professional training. The training covered the typical clinical presentations, pathophysiology, treatment plans, and key points of treatment responses for acute appendicitis (e.g., the correct treatment plan leads to improvement; the wrong treatment plan leads to worsening). After training, an assessment was conducted, and the two best-performing SPs were selected to participate in this study.

Implementation of Teaching

The internship teaching content was about appendiceal diseases from the ninth edition of Surgery, led by two experienced attending physicians from our department. These instructors, who have shown comparable performance in previous teaching evaluations and have been uniformly trained, are proficient in both SP-based scenario teaching and TT models. Three days before the internship began, the instructors shared standardized MOOC (Massive Open Online Course) videos of appendiceal disease theory, teaching rounds, and case discussion demonstrations through a WeChat group and supervised the students’ completion of self-study tasks.

TT Group: Before the internship began, instructors explained important points to the students, including medical humanities care, history taking, and examination techniques, and observed the students’ practical operations to provide immediate corrections and demonstrations. Afterward, case discussions were conducted in the classroom, where students discussed actual patient diagnoses, differential diagnoses, and treatment plans in groups and then presented their findings. Instructors were responsible for guiding the case discussions, commenting on student performance, and focusing on explaining weaknesses in students’ knowledge.

CT Group: Building on traditional internship precautions, this group emphasized the rules of scenario enactment. The SPs completed a series of diagnostic and treatment steps, such as outpatient visits, examinations, hospitalizations, treatment plan formulation, observation of condition changes, and discharge, all while questioning the physician about the condition and showing appropriate emotional responses based on the correctness of the treatment plan. To cultivate clinical thinking, SPs were also required to simulate postoperative complications, such as bleeding, residual abdominal infection, and appendiceal stump fistula. Throughout this process, the instructor provided comprehensive supervision and necessary guidance (e.g., providing examination results required by students and describing surgical procedures) to assist in student decision-making. After the diagnostic and treatment activities, the instructor evaluated the students’ performance and focused on explaining the weak points in their knowledge.

Educational evaluation

The educational evaluation section includes three main components.

Theoretical Test

Before and after the internship, students were required to complete a theoretical test through Questionnaire Star, covering clinical manifestations, diagnosis, and treatment of appendicitis, consisting of 10 questions, six of which were single-choice questions and four of which were multiple-choice questions. The full score was 100, and each student was required to spend at least 2 minutes completing the test.

Satisfaction Survey

A homemade anonymous questionnaire survey was conducted to assess student satisfaction with the two teaching methods. The questionnaire included seven questions aimed at evaluating whether teaching methods can stimulate students learning interest; enhance their clinical diagnosis and treatment capabilities, doctor‒patient communication skills, and teamwork abilities; enhance their sense of medical mission and responsibility; and facilitate the application of learned knowledge. The questionnaire was distributed and collected at the end of the course for analysis.

Case Writing Score

All students were required to complete a detailed case record based on the results of the consultation. Two professors from the research team scored the cases based on the quality of the case writing, and the average score was used as the final grade for the students.

Statistical methods

SPSS 25.0 (Armonk, NY: IBM Corp) was used for the statistical analysis. Measurement data are presented as the mean and standard deviation and were analyzed using the independent sample t-test; count data are expressed as percentages and were analyzed using the chi-square test. P<0.05 indicated a statistically significant difference.

## Results

Baseline characteristics

There were 108 students in the CT group, including 59 males and 49 females, with an average age of 21.6±0.9 years. The average score for the surgery final exam in the last semester was 72.2±4.7. There were 104 students in the TT group, including 56 males and 48 females, with an average age of 21.6±0.80 years. The average score for the surgery final exam in the last semester was 73.0±5.4. There were no statistically significant differences between the two groups of students in terms of gender, age, average score of the last semester’s final exam, or pre-internship scores (Table 1).

**Table 1:** Comparison of the study groups. CTG: Combined Teaching Group; TTG: traditional teaching group; SFEC: semester’s final exam score

	CTG (N=108)	TTG (N=104)	t	P-value
Year (x±s)	21.6±0.9	21.6±0.80	-0.119	0.905
Sex			0.13	0.909
Male	59 (62.1)	56 (49.2)		
Female	49 (37.9)	48 (50.8)		
SFEC (x±s)	72.2±4.7	73.0±5.4	1.195	0.234
Pre-internship scores (x±s)	69.4±7.3	68.7±6.7	0.823	0.412

Course outcomes

The post-internship theoretical test scores were significantly greater in the CT group (86.1±7.0) than in the TT group (79.2±6.7). Moreover, in terms of score improvement after the internship (P<0.001) and the quality of case writing (P<0.001), the CT group also performed significantly better than the TT group (Table 2).

**Table 2:** Comparison of outcomes. CTG: Combined Teaching Group, TTG: traditional teaching group

	CTG (N=108)	TTG (N=104)	t	P-value
Post-internship scores	86.1±7.0	79.2±6.7	7.28	<0.001
Improvement scores	16.6±3.7	10.6±3.8	11.72	<0.001
Quality of case writing	93.2±2.7	92.2±2.3	3	0.003

Satisfaction surveys

A total of 212 satisfaction surveys were distributed and collected, all of which were valid. In terms of stimulating learning interest, enhancing clinical diagnostic and therapeutic capabilities, improving doctor-patient communication skills, strengthening the sense of medical mission, enhancing the sense of physician responsibility, and facilitating the application of learned knowledge, the proportion of students who agreed was significantly greater in the CT group than in the TT group (P<0.05). There were no significant differences between the two groups in terms of team collaboration skills (P=0.133), and the specific results are detailed in Table 3.

**Table 3:** Satisfaction survey for general surgery clinical clerkship.

	CTG (N=108)	TTG (N=104)		P-value
stimulating learning interest			5.947	0.015
Yes	103 (94.8)	89 (81)		
No	5 (5.2)	15 (19)		
Enhanced clinical capabilities			35.77	<0.001
Yes	105 (97)	68 (60)		
No	3 (3)	36 (40)		
Improved doctor-patient communication skills			67.31	<0.001
Yes	106 (98)	45 (49)		
No	2 (2)	59 (51)		
Enhanced team collaboration skills			2.25	0.133
Yes	104 (95)	95 (90)		
No	4 (5)	9 (10)		
Strengthened medical responsibility			22.93	<0.001
Yes	108 (100)	84 (75)		
No	0	20 (25)		
Strengthened medical mission			24.21	<0.001
Yes	108 (100)	83 (81)		
No	0	21 (19)		
Beneficial for the application of learned knowledge			67.66	<0.001
Yes	108 (100)	60 (70)		
No	0	44 (30)		

## Discussion

This study innovatively combines SP and scenario-based teaching methods, applying a scenario-based teaching model in the whole diagnosis and treatment process with SPs in the clinical internship of general surgery [9,10]. The results of a prospective randomized single-blind controlled study showed that scenario-based teaching with SPs outperforms TT methods in terms of post-internship theoretical scores and the quality of medical record writing [11,12]. Additionally, in terms of stimulating students’ learning interest; enhancing clinical diagnostic and therapeutic abilities, doctor‒patient communication skills, and teamwork abilities; strengthening the sense of medical mission and physician responsibility; and facilitating the application of acquired knowledge to real-world situations, the CT method was significantly more effective than TT method [13-15]. This suggests that the scenario-based teaching model with SPs creates a teaching scenario closer to the real medical environment, better-cultivating students’ clinical skills and humanistic qualities and proving to be more effective in clinical internships than TT.

SPs are a new type of simulated patient characterized by their ability to mimic the progression and reactions of real patients, thus integrating academic knowledge with clinical practice [16,17]. They have been widely used in clinical internships and have achieved good teaching results [18,19]. The combined application of SPs with other teaching methods, such as PBL, CBL, and mind mapping, has also further improved teaching effectiveness [20,21]. In our CT method practice, we found students inquiries into the medical history of SPs are more systematic and comprehensive, avoiding concerns about patients' concealment of medical history or noncooperation from family members, thereby improving the completeness of students' diagnostic and treatment skills. However, previous studies have mostly utilized SPs for medical history inquiries, physical examinations, and doctor‒patient communication without providing rational feedback on students’ treatment plans [22]. Consequently, students are unable to obtain sufficient diagnostic and therapeutic feedback, cannot experience the emotional facets of the diagnostic process, and find it difficult to understand their shortcomings and areas for improvement after learning. Additionally, there is a lack of medical ethics and professional conduct, which limits their ability to train students in clinical thinking, clinical decision-making, and humanistic qualities [23,24].

In our study, the use of SPs goes beyond simple role-play. SPs, trained by experts, provide feedback based on the correctness of the intern doctors’ treatment plans. This requires more from SPs than just their role. Unlike role play, where individuals may improvise their responses, SPs adhere to a detailed script that outlines the patient's history, examination findings, and emotional responses. This script is developed by medical professionals to ensure the portrayal is medically accurate and educationally relevant. The primary goal is to provide students with a realistic clinical experience that allows for the assessment and enhancement of their diagnostic skills, communication abilities, and professional behaviors in a safe and controlled setting.

Through rigorous training of SPs and the method of teachers acting as SPs, the internship process recreated real diagnostic and treatment scenarios through virtual scenario teaching [7,25]. Based on a SPs’ comprehensive understanding of the portrayed diseases, the diagnostic and treatment plans made by students could receive timely and correct feedback [26]. Patients’ emotional changes and appropriate doctor‒patient communication allowed them to experience the challenges they might face as real physicians [27]. Through rich emotional experiences, students gained comprehensive psychological development. Our satisfaction survey indicated that students are highly interested in the scenario-based teaching model with SPs [9,28]. This may be because this teaching model allows students to experience the significance of being a doctor-feeling the joy of using their knowledge to alleviate patients’ suffering and the pain caused to patients by improper diagnosis and treatment [29]. This sense of urgency inspired deep respect for life in students, thereby more effectively cultivating their sense of medical responsibility and mission [30]. Repeated discussions during the diagnostic process not only strengthened students’ team awareness but also, through discussion, promoted the interaction and collision of thoughts among students, further broadening the team’s knowledge domain and cultivating their team spirit and clinical diagnostic and therapeutic capabilities, which is also very beneficial for applying learned knowledge to clinical practice, as confirmed in the satisfaction survey [31].

Moreover, timely feedback from SPs on students’ treatment plans is crucial for improving students’ clinical capabilities [32]. Correct treatment plans relieve patients' symptoms, consolidating the internalization of theoretical knowledge and the application of diagnostic and treatment strategies [33]. In contrast, incorrect treatment plans exacerbate patients' symptoms, prompting students to reflect and adjust their treatment plans in a timely manner [34]. This multidimensional teaching model greatly enhances students’ clinical decision-making abilities and problem-solving skills, and through emotional experiences in the diagnostic process, it cultivates students’ sense of mission and responsibility [35]. This positive and negative feedback is very beneficial for the formation of students’ knowledge structures, as evidenced by the performance of the integrated group in both theoretical scores and medical record writing quality [36]. These results indicate that the scenario-based teaching model with SPs (CT) is superior to TT methods and deserves further promotion.

Limitations

First, the performance and feedback of SPs depend entirely on the quality and intensity of prior training; if they could not accurately simulate real patients' conditions, the effectiveness of the internship would be affected. Second, the scenario-based teaching model requires a higher level of organizational management and a real-time feedback system, posing greater demands on teaching resources and instructors, which not all educational environments can meet. Additionally, the methods for assessing learning outcomes need to be more scientific and comprehensive to ensure accurate evaluation of learning effects across various dimensions. Therefore, future research should attempt to verify the conclusions of this study through multicenter, large-sample studies and improve and refine the scenario-based teaching model with SPs to better align with real clinical environments and high-quality teaching needs. Overall, this study confirms that the application of the scenario-based teaching model with SPs in general surgery internships can significantly improve students' theoretical knowledge levels and clinical skills while promoting the comprehensive development of medical students, including professional skills, humanistic care, and professional responsibility. Future educational practices can continue to explore the application effects and optimization paths of this teaching model in other clinical subjects, bringing broader development prospects to medical education.

## Conclusions

This study validates the substantial utility of the patient-centered scenario simulation method in general surgery training. It improves students' theoretical and clinical competencies and fosters holistic development in professional skills, compassionate care, and ethical responsibility. Consequently, medical education should persist in refining and advancing this teaching approach, aligning it with evolving healthcare and educational demands to nurture exceptional medical professionals.
